# Variability in Immunohistochemical Detection of Programmed Death Ligand 1 (PD-L1) in Cancer Tissue Types

**DOI:** 10.3390/ijms17050790

**Published:** 2016-05-21

**Authors:** Giosuè Scognamiglio, Anna De Chiara, Maurizio Di Bonito, Fabiana Tatangelo, Nunzia Simona Losito, Annamaria Anniciello, Rossella De Cecio, Crescenzo D’Alterio, Stefania Scala, Monica Cantile, Gerardo Botti

**Affiliations:** 1 Pathology Unit, Istituto Nazionale Tumori Fondazione “G. Pascale”, via Mariano Semmola, 80131 Napoli, Italy; giosco80@gmail.com (G.S.); a.dechiara@istitutotumori.na.it (A.D.C.); mauriziodibonito@libero.it (M.D.B.); f.tatangelo@istitutotumori.na.it (F.T.); n.losito@istitutotumori.na.it (N.S.L.); a.anniciello@istitutotumori.na.it (A.A.); r.dececio@istitutotumori.na.it (R.D.C.); g.botti@istitutotumori.na.it (G.B.); 2 Molecular Immunology and Immunoregulation Functional Genomics, Istituto Nazionale Tumori Fondazione “G. Pascale”, via Mariano Semmola, 80131 Napoli, Italy; c.dalterio@istitutotumori.na.it (C.D.); s.scala@istitutotumori.na.it (S.S.)

**Keywords:** PD-L1, immunohistochemical staining, tissue type’s variability

## Abstract

In normal cell physiology, programmed death 1 (PD-1) and its ligand, PD-L1, play an immunoregulatory role in T-cell activation, tolerance, and immune-mediated tissue damage. The PD-1/PD-L1 pathway also plays a critical role in immune escape of tumor cells and has been demonstrated to correlate with a poor prognosis of patients with several types of cancer. However, recent reports have revealed that the immunohistochemical (IHC) expression of the PD-L1 in tumor cells is not uniform for the use of different antibodies clones, with variable specificity, often doubtful topographical localization, and with a score not uniquely defined. The purpose of this study was to analyze the IHC expression of PD-L1 on a large series of several human tumors to correctly define its staining in different tumor tissues.

## 1. Introduction

Programmed death 1 ligand (PD-1) is a 40 kDa transmembrane protein that is expressed on a large series of normal tissues including epithelial cells, vascular endothelial cells, natural killer cells, macrophages, myeloid dendritic cells and B cells [1]. During the normal cell physiology, programmed death 1 (PD-1) is activated by binding with two ligands, PD-L1 and PD-L2. The binding with PD-L1, expressed on the surface of activated cytotoxic T cells, inhibits Interleukin 2 (IL-2) production and T cell activation reducing phosphorylation of ZAP70 and PKC θ [2]. PD-1/PD-L1 interaction represents a crucial regulatory check against an enormous adoptive immune response to antigens and autoimmunity [3].

Expression of PD-L1 in donor tissue was essential for the prevention of chronic rejection in a heart transplant model [4] and in the support of tolerance at the utero–placental interface [5].

Several studies have recently shown that PD-1/PD-L1 pathway may have a key role in the interaction of tumor cells with host immune response, and tumor cells PD-L1 expression may function as a mechanism of adaptive immune resistance. PD-1 was detected in tumor-infiltrating lymphocytes (TILs), present in tumor microenvironment, and its aberrant expression was associated to a poor prognosis in several human tumors. Moreover, many human cancers, including stomach, breast, ovarian, renal, melanoma, pancreatic and lung cancers, have been shown to express PD-L1 and, in most of cases, its expression was correlated with a poor prognosis [6,7,8,9,10].

The correlation between PD-1/PD-L1 expression and worse patient outcomes supports the hypothesis that these molecules can represent potential prognostic biomarkers in many human solid tumors.

Early-phase trials using monoclonal antibodies targeting PD-1 or PD-L1 revealed a real efficacy in clinical response in patients with refractory tumors [11]. For this reason, many studies are also evaluating the predictive value of this marker, mainly through its detection with *in situ* methods, such as immunohistochemistry and mRNA *in situ* hybridization [12,13].

However, the data available in literature, related to the expression of PD-L1 in different tumor types, are not uniform and are often conflicting. This problem might be associated with the use of different antibodies clones, with variable specificity, and mainly with a score not uniquely defined.

Recently the FDA has approved several PD-L1 antibody clones for diagnostic use in Non-Small Cell Lung Cancer (NSCLC), but their validation on different tumor types and especially the definition of threshold in terms of the percentage of positive cells is still in progress [14].

For this reason, to reduce the subjectivity of the interpretation and in order to properly stratify patients to specific immunotherapies, the expression PD-L1 should be better explored in large series of human tumors also to define potential difference associated to different cell-types.

In this study we analyzed PD-L1 expression in a series of solid and hematological human neoplasm, such as breast, ovarian, colon, kidney, thyroid, lung, melanoma, sarcoma tumor and lymphomas, to define a standardized protocol for immunohistochemistry analysis and to establish the correct and specific evaluation parameters for different tumor types.

## 2. Results

### 2.1. Development and Validation of Immunohistochemistry Assay for Programmed Death Ligand 1 (PD-1) Detection

**Figure 1:**
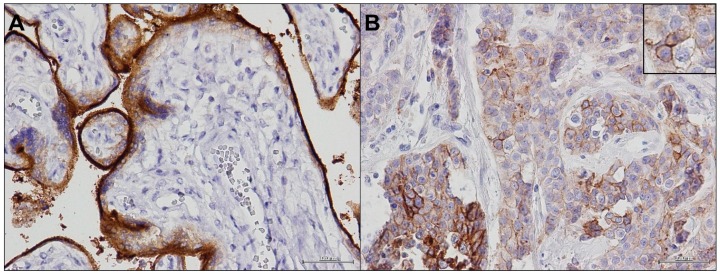
PD-L1 (programmed death ligand 1) staining: (**A**) Anti-PD-L1 (SP-142) clone in placenta sample (40×); and (**B**) Anti-PD-L1 (SP-142) clone in tumor sample (40×) with a detail of cell membrane positivity (60×).

To realize our experimental purpose, we used one of the four antibody clones approved by FDA for PD-L1 immunohistochemical (IHC) detection, the Rabbit monoclonal antibody (mAb) Anti-PD-L1 (SP-142) clone. We tested it with different pre-treatments, antibody concentrations, and detection reagents to optimize the conditions to improve the performance of each antibody. The validation of PD-L1 staining was carried out on placenta tissues as positive control (Figure 1A) and on selected tumor samples (Figure 1B).

SP-142 Ab clone detected membrane isoforms of PD-L1. It was highly expressed in placenta cells and with a variable intensity in positive tumor cells.

### 2.2. PD-L1 Immunohistochemical (IHC) Expression in Epithelial Cancers

We selected 20 breast cancer samples with different histology, stage and grading. Our results showed a heterogeneous expression of PD-L1 on breast tumor cells.

For its assessment, we considered both qualitative and quantitative parameter. For the qualitative criteria, we considered the immunoreactivity of membrane dividing it into “absent”, “incomplete” and “complete”, and the intensity of the reaction at the membrane level, dividing it into “mild”, “moderate” and “intense”. In some cases, we also detected a mild cytoplasmic expression. For the quantitative criteria we considered the percentage of positive tumor cells ≥1%.

**Figure 2:**
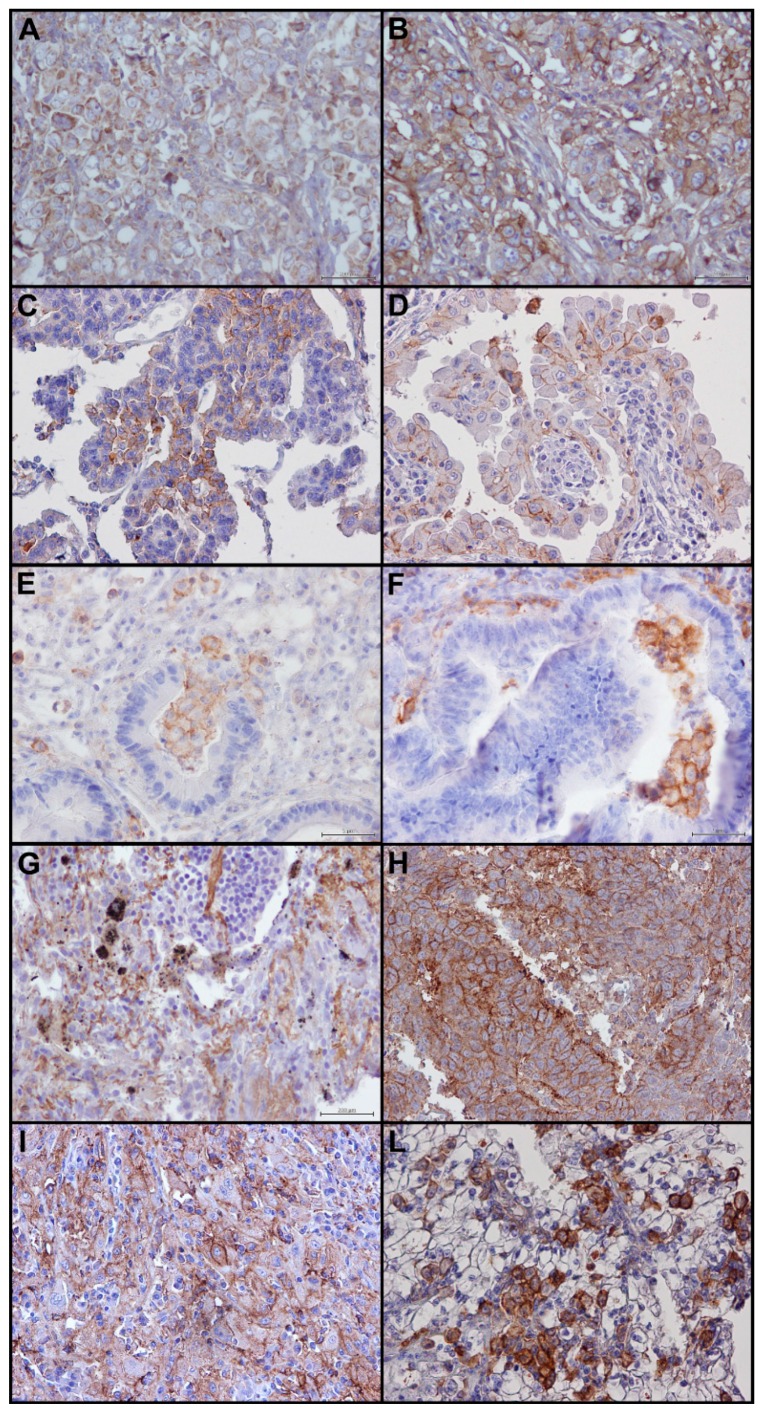

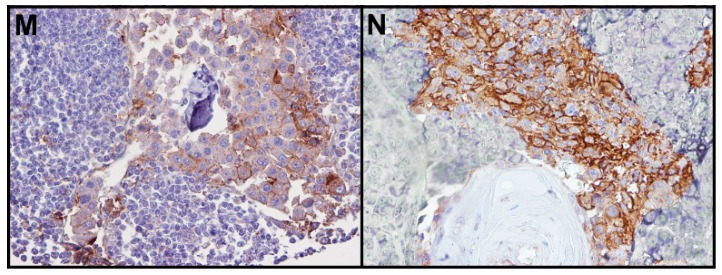
PD-L1 staining in epithelial tumors: (**A**) incomplete immunoreactivity of membrane in breast cancer sample (40×); (**B**) complete immunoreactivity of membrane in breast cancer sample (40×); (**C**) incomplete immunoreactivity of membrane in ovarian cancer sample without cytoplasmic staining (40×); (**D**) incomplete immunoreactivity of membrane in thyroid cancer sample with mild cytoplasmic staining (40×); (**E**) incomplete immunoreactivity of membrane in colon cancer sample (40×); (**F**) complete immunoreactivity of membrane in colon cancer sample (40×); (**G**) incomplete immunoreactivity of membrane with mild cytoplasmic staining in lung cancer sample (40×); (**H**) complete immunoreactivity of membrane with mild cytoplasmic staining in lung cancer sample (40×); (**I**) incomplete immunoreactivity of membrane without cytoplasmic staining in kidney cancer sample (40×); (**L**) complete immunoreactivity of membrane with cytoplasmic staining in kidney cancer sample (40×); (**M**) incomplete immunoreactivity of membrane in melanoma sample (40×); and (**N**) complete immunoreactivity of membrane in melanoma sample (40×).

We detected PD-L1 expression in about 60% of samples with a prevalent moderate/intense expression in ≥10% of tumor cells, in 20% of cases with an incomplete (Figure 2A) and in 40% of cases with a complete immunoreactivity of membrane (Figure 2B), with and without a mild cytoplasmic staining.

Similar to breast cancer, in ovarian cancers, the percentage of samples showing positive staining for PD-L1 was low, and it was present in a few cell aggregates. PD-L1 expression showed a prevalent moderate expression in ≥1% of tumor cells. In all positive cases, an incomplete immunoreactivity of membrane was detected, in some cases with and without cytoplasmic staining (Figure 2C).

In selected thyroid cancers, with different histology, we detected PD-L1 expression in very few cases, <10% of samples. For the quantitative criteria, we considered the percentage of positive tumor cells ≥1%.

In positive samples, we highlighted only an incomplete immunoreactivity of membrane without cytoplasmic staining (Figure 2D), with few cells, in the same area, with a complete immunoreactivity of membrane.

In colon cancer, the percentage of samples with a positive staining for PD-L1 is less than 10%. All positive specimens showed an incomplete (Figure 2E) and in very few cell aggregates a complete immunoreactivity of membrane (Figure 2F). PD-L1 expression in ≥1% of tumor cells identified positive samples.

We selected 20 non-small cell lung cancers (NSCL), in which we detected PD-L1 expression in about 10% of samples with a prevalent moderate expression in ≥1% of tumor cells. In the positive samples, we highlighted a complete immunoreactivity of membrane with a mild cytoplasmic staining (Figure 2G), but an incomplete immunoreactivity of membrane was also present (Figure 2H).

In selected kidney cancers, we detected PD-L1 expression in about 20% of samples with a prevalent moderate expression in ≥10% of tumor cells. In the most of positive samples we highlighted an incomplete immunoreactivity of membrane (Figure 2I) and in few case a complete immunoreactivity of membrane (Figure 2L) with or without a mild cytoplasmic staining.

In the 20 selected melanoma samples, the pattern of interpretation of PD-L1 was more complex.

We detected PD-L1 expression in about 60% of samples with a heterogeneous expression. For the quantitative criteria, we considered the percentage of positive tumor cells ≥1%. In most of the cases, an incomplete immunoreactivity of membrane was detected with or without a mild cytoplasmic expression (Figure 2M). In few positive samples, we highlighted a complete immunoreactivity of membrane with or without a mild cytoplasmic staining (Figure 2N). In some samples, TIL component was negative for PD-L1, while in most of the positive samples TIL component showed a positive staining for PD-L1.

### 2.3. PD-L1 IHC Expression in Soft Tissue Tumors

We selected 20 soft tissue tumors, mainly angiosarcomas, to evaluate PD-L1 staining in mesenchymal tumors.

**Figure 3:**
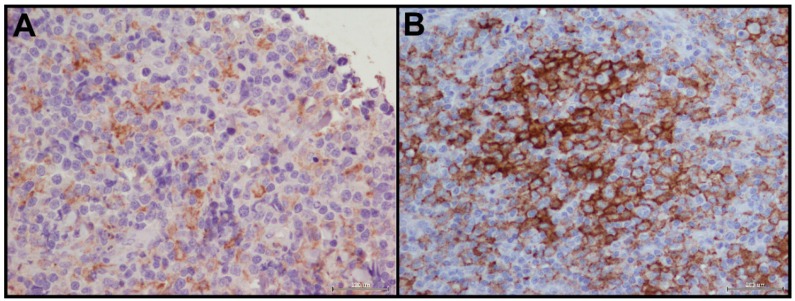
PD-L1 staining in soft tissue tumors: (**A**) incomplete immunoreactivity of membrane (40×); and (**B**) complete immunoreactivity of membrane (40×).

We detected PD-L1 expression in 50% of samples with a prevalent moderate expression in ≥1% of tumor cells, in 35% of cases with an incomplete (Figure 3A) and only in 15% of cases with a complete immunoreactivity of membrane (Figure 3B).

### 2.4. PD-L1 IHC Expression in Non-Hodgkin Lymphoma

**Figure 4:**
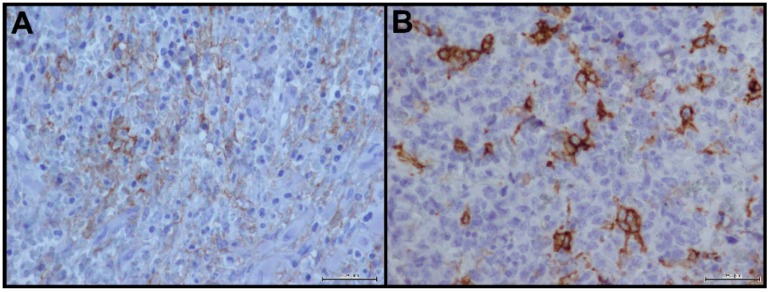
PD-L1 staining in non-Hodgkin lymphoma: (**A**) incomplete immunoreactivity of membrane (40×); and (**B**) complete immunoreactivity of membrane (40×).

Selected non-Hodgkin lymphoma samples showed a consistent expression of PD-L1 in 60%–70% of tumor samples with a clear incomplete (Figure 4A) and complete (Figure 4B) immunoreactivity of membrane in ≥10% of tumor cells.

All data of PD-L1 IHC staining criteria for each tumor type are schematized in Table 1.

## 3. Discussion

Targeting immune checkpoints such as PD-1, PD-L1 and cytotoxic T lymphocyte antigen 4 (CTLA 4) have shown to have a great therapeutic value in different tumors revolutionizing the scenario of treatment strategies [15]. PD-1 or PD-L1 inhibitors administrated as single agents have resulted in durable tumor regression in some patients [16]. However, it is not yet clear whether the expression of these markers on the membrane of tumor cells and lymphocytes in the tumor microenvironment may be useful in the correct stratification of patients to address toward specific immunotherapies.

At present, treatment regimens contemplate the use of PD-1/PD-L1 inhibitors regardless of their *in vivo* expression. However, the inclusion in a diagnostic report of a value associated with PD-L1 expression on tumor cells could be a useful tool for oncologists to determine which patients may be more responsive to specific therapies. Discordant data about the prognostic value of PD-L1 tumor cells-associated expression in cancer have been debated in the literature. The scenario appeared complex, mainly for the great difficulty in achieving accurate and reproducible assessment of PD-L1 expression across different tumor types.

Despite the well-known potential of PD-L1 as prognostic and predictive biomarker, few studies describing its IHC expression in cancer subtypes are currently present in the literature. This could be due to the extreme difficulty in developing a standard protocol for different commercialized antibodies, which often recognize different isoforms of PD-L1, and also because the cut-off values for its correct interpretation have not yet been completely defined.

Recently, the FDA has approved several PD-L1 antibody clones for diagnostic use in NSCLC but their validation on different tumor types and especially the definition of threshold in terms of the percentage of positive cells is still in progress.

In this study, we used one of the FDA-approved antibody clones, and selected a discrete series of different human tumors to perform PD-L1 protein expression by immunohistochemistry and define a detailed operative protocol. Moreover, we established and suggested a score system for several tumors, normalizing PD-L1 IHC expression in a series of 20 samples for each tumor type.

Overall, our data highlighted a heterogeneous expression of PD-L1 in different tumor tissues analyzed.

In breast cancer tissue samples, we detected a more consistent staining of PD-L1 in tumor cells, particularly those near to the front of inflammatory cells. The complete and incomplete positivity membrane could certainly be one of the parameters to consider in order to properly define the prognostic value of the marker in tumor patients.

In the literature, only few papers have described PD-L1 expression in breast cancer subtypes and in most cases the studies were conducted on cell models and through molecular analysis [10,17].

Only few studies analyzed PD-L1 expression by immunohistochemistry in breast cancer. Muenst S examined IHC PD-L1 expression in a case series of 650 breast cancer samples highlighting that its expression was significantly associated with age, tumor size, lymph node status and worse overall survival (OS). IHC results showed in all cases a strong cytoplasmic positivity that made its interpretation difficult and unclear. Finally, on a case series of 161 triple negative breast cancer patients, where the authors have also considered stromal and cytoplasmic positivity, the only cytoplasmic expression of PD-L1 was associated with a lower risk of breast cancer death, without clarifying and defining the real value of this positivity in tumor cells [18].

In ovarian cancer, we detected a low expression of PD-L1, always with an incomplete immunoreactivity of membrane.

Very few studies described IHC expression in ovarian cancer subtypes. First studies carried out on FFPE samples with an “in house” antibody against PD-L1, showed a strong correlation between its expression and poorer prognosis [19]. More recently, Abiko *et al.* analyzed IHC expression of PD-L1 in a series of 27 ovarian cancer in the same condition of Hamanishi *et al.* suggesting a potential evaluation score, but without detailing accurately the staining in tumor cells [20].

In renal carcinoma samples, we detected PD-L1 expression with a complete/incomplete immunoreactivity of membrane.

In the literature, only few papers describe immunohistochemical expression of PD-L1 [21,22] in kidney cancer, showing an association between its upregulation and shorter survival in patients with metastatic Renal Cell Carcinoma (RCC). The evaluation score of PD-L1 was similar to that highlighted by us, but also in this case the cytoplasmic positivity appeared prevalent.

In colon cancer, we detected PD-L1 expression in few cases with a very similar staining to breast cancer cells, with a complete or incomplete immunoreactivity of membrane.

PD-L1 expression was previously analyzed in a TMA containing a large series of colorectal cancers (CRC) with two PD-L1 specific antibody preparations (monoclonal and polyclonal ab). Although the two procedures were fairly superposable, in this case the cytoplasmic positivity was also prevalent [23].

In melanoma, we detected PD-L1 expression in 10% of tumor samples with a heterogeneous panel of staining.

More consistent literature describes the role of PD-L1 in melanoma because the specific therapies directed against PD-1/PD-L1 pathway are also more effective and already operative in metastatic melanoma patients.

Regarding PD-L1 IHC detection in melanoma tumor cells, a recent study showed a clear membrane positivity in about 37% of patients with a correlation with tumor progression and poor survival [24,25].

In selected thyroid cancer, we detected PD-L1 expression in very few cases with a prevalent incomplete immunoreactivity of membrane.

The only one study analyzing PD-L1 IHC expression in thyroid cancer cells again showed a prevalent cytoplasmic expression [26].

Finally, in lung cancer (NSCLC), we detected PD-L1 a complete immunoreactivity of membrane.

Abundant literature is present on PD-L1 role in lung cancer and several studies have described in detail its IHC expression in tumor cells [27,28].

In selected sarcoma samples, mainly represented by angiosarcomas, we detected PD-L1 tumor expression with a milder expression than other epithelial tumors.

Also in this case, few papers are present in the literature on the role of PD-L1 in soft tissue sarcoma (STS). Kim *et al.* evaluated PDL1 expression in 105 STS highlighting its prognostic value [29] while more recently discordant data were described [30].

Our non-Hodgkin lymphoma samples showed a consistent expression of PD-L1 with a clear incomplete and complete immunoreactivity of membrane.

Several studies described PD-L1 IHC expression in lymphoma cells, in particular its aberrant expression was detected in some aggressive B-cell lymphomas and virus- and immunodeficiency-associated tumors associated with an ineffective T-cell immune [31].

## 4. Materials and Methods

### 4.1. Patients Selection

One hundred eighty oncologic patients that underwent surgery at the National Cancer Institute “Giovanni Pascale Foundation” of Naples, Italy, were enrolled into this study. In detail, we selected 20 breast cancer, 20 ovarian cancer, 20 renal cancer, 20 colon cancer, 20 malignant melanoma, 20 lung cancer, 20 thyroid cancer and 20 sarcoma tumors, and 20 non-Hodgkin lymphoma. A placenta tissue sample was included in our series to perform PD-L1 staining.

All cases of tumor samples were reviewed according to specific World Health Organization (WHO) classification criteria, using standard tissue sections and appropriate immunohistochemical slides.

Medical records for all cases were reviewed for clinical information, including histologic parameters that were determined from the Hematoxylin & Eosin (H&E) slides. The following clinical and pathological parameters were evaluated for each tumor included in the study: patient age at initial diagnosis, tumor size, histologic subtype, histologic grade, nuclear grade, nodal status, number of positive lymph nodes, tumor stage, tumor recurrence or distant metastasis and type of surgery (for tumor removal).

In addition, all specimens were characterized for all routinely diagnostic immunophenotypic parameters.

### 4.2. Immunohistochemical Analysis

#### 4.2.1. PD-L1 Immunohistochemistry Assay Optimization

FFPE placenta samples were used for final assay optimization. As part of the optimization process, we tested SP-142 antibody concentration, antibody incubation time, antigen retrieval reagents and methods, and antibody detection system. SP-142 antibody concentrations ranged from 0.075 to 4.5 μg/mL, and the incubation times ranged from 1 h to overnight (14 h). Multiple antigen retrieval methods were tested.

Other than protein block and primary antibody diluent (Bond primary antibody diluent, #AR9352, Leicabiosystems, Newcastle, UK), all tested reagents are commercially available.

#### 4.2.2. Optimized PD-L1 IHC Protocol

Tissue processing: FFPE tumor tissue sections of 3–4 μm thickness were cut onto adhesive slides (#KP-SIL-3056, KP-Silan adhesive slides, Klinipath BV, Typograaf, Duiven, The Netherlands) baked at 65 °C (dry heat) for 1 h less than 1 week before use, deparaffinized in four changes of 100% xylene and rehydrated with a graded ethanol series (100%, 70%, and 40%) to distilled water.

Prepared slides were incubated for 12 min at 110 °C in Cell Conditioning Solution 1 (Ventana medical Systems (Cat #: 950-124), Tucson, AZ, USA), using a commercial steamer as heat source (Biocare Medical, Decloaking Chamber DC12, Pike Lane Concord, CA, USA). After cooling for 20 min, automatic staining was performed.

After cooling for 20 min, automated staining was performed using an automated IHC staining platform (DAKO autostainer Link48, Glostrup, Denmark).

All procedures were carried out at room temperature (circa 25 °C) following a 5-min incubation with a peroxidase blocking reagent (DAKO #SM801, Glostrup, Denmark) and following a 5-min incubation with a protein serum block (1% goat serum (bcam Cambridge Science Park Milton Road, Cambridge, UK), 4% BSA in PBS, slides were incubated with the anti-PD-L1 antibody clone SP-142 (Spring Bioscience (M4420) Koll Center Pkwy, Pleasanton, CA, USA) at a concentration of 3.75 μg/mL in a primary antibody diluent (Bond primary antibody diluent, #AR9352, leicabiosystems, Newcastle, UK) for 90 min.

The Goat Anti-Rabbit + HRP (horseradish peroxidase) visualization reagent (DAKO, Cat #SK001, Glostrup, Denmark) was used for primary antibody detection.

The secondary antibody was incubate for 40 min, following incubation with DAB substrate buffer + DAB chromogen (3,3′-diaminobenzidine) (DAKO, SK001). During incubations, slides were washed with Bond Wash Solution (DAKO, SK310). The slides were counterstained on platform with hematoxylin (DAKO, K8008), and rinsed in distilled water. The slides were dehydrated out of platform in an ethanol series (30%, 70%, and 100%) and four changes of 100% xylene, and permanently sealed with coverslips in automatic (DAKO #CS100, Glostrup, Denmark).

## 5. Conclusions

A correct standardization of IHC protocols for the detection of PD-L1 in tumor cells in different cancer subtypes and a definition of an adequate cut-off in terms of percentage of positive cells will enable reevaluating the prognostic value of this marker, but especially highlighting its predictive value for the therapeutic stratification of cancer patients.

Unfortunately, preliminary data would seem to indicate that patients who can better benefit of from PD-1/PD-L1 therapy inhibitors are those with positive staining of PD-L1 on tumor cell surface [32]. Therefore, the validation of these data through standardized procedures is of crucial importance, since it would provide a precious predictive marker of response, currently not available for immune checkpoint inhibition.

## Figures and Tables

**Table 1:** PD-L1 (programmed death ligand 1) immunohistochemical (IHC) staining criteria for each tumor type.

Tumor Type	Percentage pf PD-L1 Positive Tumor Cells	PD-L1 Intensity of Reaction	PD-L1 Complete Membrane Positivity	PD-L1 Incomplete Membrane Positivity	PD-L1 Cytoplasmic Positivity
Breast cancer	60%	Moderate/Intense	+	+	+/−
Ovarian cancer	<10%	Moderate	−	+	+/−
Thyroid cancer	<10%	Mild	+	+	−
Colon cancer	<10%	Moderate	+	+	−
Lung cancer	10%	Moderate	+	+	+
Kidney cancer	20%	Moderate	+	−	+/−
Melanoma	60%	Moderate/Intense	+	+	+/−
Sarcoma	50%	Moderate	+	+	+/−
Non-Hodgkin lymphoma	60%	Moderate/Intense	+	+	−
